# Differences in Pre-Laying Behavior between Floor-Laying and Nest-Laying Pekin Ducks

**DOI:** 10.3390/ani9020040

**Published:** 2019-01-29

**Authors:** Lorelle Barrett, Irek Malecki, Dominique Blache

**Affiliations:** 1 School of Agriculture and Environment, The University of Western Australia, Perth, WA 6009, Australia; dominique.blache@uwa.edu.au; 2 Poultry CRC, University of New England, Armidale, NSW 2351, Australia; 3 Milne AgriGroup Ltd., Welshpool, Perth, WA 6106, Australia; imalecki@iinet.net.au

**Keywords:** Pekin duck, floor laying, nesting behavior, competition

## Abstract

**Simple Summary:**

Floor-laying is a behavior seen in farmed ducks, where eggs are laid onto the shed floor rather than in the nest boxes provided. This behavior costs producers, due to damaged eggs, and may be a negative experience for those birds performing it. However, factors contributing to floor laying in Pekin ducks are not well understood. We aimed to identify whether behavioral differences exist between floor and nest-laying ducks. We found that some floor-laying ducks never interacted with nest boxes, while other floor-layers used nests in a similar way to nest-laying ducks. Floor-layers that did not interact with nests experienced less aggression than the other two groups. We concluded that competition is a contributing factor to floor-laying in ducks, and it is possible that those ducks not using nests avoid doing so to reduce agonistic encounters. Developing strategies to reduce competition for nest boxes may help improve production efficiency and minimize negative welfare outcomes that might exist for floor-laying ducks.

**Abstract:**

Floor-laying in commercially farmed Pekin ducks is not well understood. This exploratory study aimed to determine if behavioral differences exist between floor-laying and nest-laying ducks. Retrospective analysis of video footage from a small commercial breeding flock (n = 60 birds) was used to quantify the behavior of floor-laying and nest-laying birds (n = 24 events per group) in the hour prior to oviposition site selection. The frequency, percentage of time spent, and duration of bouts were compared for nest box interactions, behaviors inside and outside of boxes and aggressive interactions. Some floor-laying birds did not enter or investigate nest boxes (FL-Out), whilst some floor-layers (FL-In) used nest boxes similarly to nest-laying birds (NL). Nest-building behavior differed only in location, with FL-Out performing the behavior on the shed floor and the other groups performing it primarily in boxes. FL-Out sat more, walked less, and engaged in less aggression (*p* < 0.05) than FL-In and NL. The occurrence of multiple birds in a nest box was strongly correlated with the number of aggressive interactions that occurred in the box (R = 0.81). Competition appears to contribute to floor-laying in Pekin ducks; FL-Out birds may not engage with nest boxes as a coping strategy to avoid agonistic behavior. These findings indicate that developing practical strategies to reduce nest box competition could help mitigate floor-laying. However, other factors such as nest design may also contribute to FL-Out birds’ reluctance to use nest boxes and require further investigation.

## 1. Introduction

In commercial Australian duck farms, breeding females are provided with substrate-lined nest boxes at floor level to encourage nesting behavior and to make manual egg collection more efficient. Despite the provision of nest boxes, eggs are frequently laid outside of these boxes on the shed floor, a behavior that is known as floor-laying. Ducks lay between 30% and 40% of their eggs on the floor in the early part of their laying cycle, before decreasing and stabilizing at around 10% [1]. In Australia, it is estimated that 20% of eggs laid in commercial farms are floor-eggs [2]. Floor-laying is undesirable because it impacts a farm’s production efficiency through lost potential income from breakages, contamination or decreased hatchability [3].

From an animal welfare perspective, it is possible that floor-laying is a negative experience for ducks. Egg-laying is hormonally regulated and results in a strong pre-laying motivation to find an appropriate nest site [4]. A bird unable to adequately perform such behavior may experience stress and negative emotions, such as frustration [5]. Domestic ducks are strongly motivated to lay eggs in an enclosed nest site, which is in keeping with the observed nesting behavior of wild Mallards [6]. Pekin ducks preferentially nest in boxes that provide a high level of concealment, and those birds that had established floor-laying due to nest box deprivation preferentially lay eggs in nest boxes once these are available, rather than continuing to lay on the floor [7]. These motivational behaviors strongly suggest that a duck that cannot lay in a suitable nest site may suffer psychologically and therefore be subjected to poorer welfare. There is some evidence in chickens to support this: in hens that habitually floor-laid even when nest boxes were available, a positive correlation was found between the proportion of floor-eggs and behaviors indicative of frustration [8].

In domestic hens, floor-laying has been associated with nest design [9,10], early experience [11,12], rearing environment [13], genetic strain [14] and competition for nest sites [14,15]. Little is known about how these factors contribute to floor-laying in ducks. Although previous studies suggest that Pekin ducks are typically very motivated to lay in suitable nest sites [7,16], persistent floor-laying (laying > 70% of eggs on the floor) has also been identified in a small percentage (5%) of ducks [16], suggesting that some ducks have little motivation to use nest boxes. Reasons for the variation in motivation to use nest boxes are yet to be fully explored in this species.

Competition appears to be a contributing factor to floor-laying in Pekin ducks, as the incidence of floor-laying decreases if the ratio of nest boxes to laying females is higher [16]. This same study also found that more than two thirds of floor-eggs were laid during the time of highest nest box use, further supporting the theory of competition. Social hierarchy appears to be related to the competition for nest sites in hens, with dominant birds receiving fewer aggressive pecks just prior to oviposition [17]. However, no study has formally assessed the effect of social factors, such as competition and hierarchy, on floor-laying in ducks.

The quantification of duck behavior prior to egg-laying would help to better understand how factors such as competition or disinterest in nest boxes contribute to the incidence of floor-laying in this species. Understanding the causes of floor-laying would subsequently help improve production efficiency and welfare through enhanced management of the behavior. The aim of this exploratory study was to quantify the behavior of ducks that floor-laid and ducks that nest-laid in the hour before oviposition site selection. Behavioral analysis of video footage of a small duck breeding flock was used to test the hypotheses that (1) pre-laying behavior is different between birds that floor-laid and birds that nest-laid, and (2) the pre-laying behavior of birds within the floor-laying population is variable.

## 2. Materials and Methods

### 2.1. Animals and Husbandry

**Figure 1:**
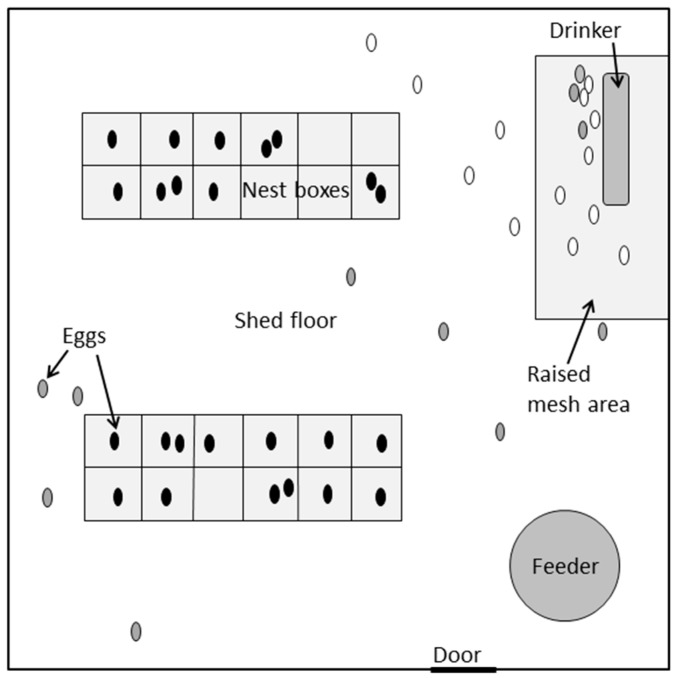
Schematic diagram of the pen layout used for video recording pre-laying behavior and the egg-laying locations of analyzed birds. Pen dimensions 5 m × 5 m. Black ovals = eggs laid in nest boxes; grey ovals = eggs laid on floor by ducks that used nest boxes; white ovals = eggs laid on the floor by ducks that did not use nest boxes.

**Figure 2:**
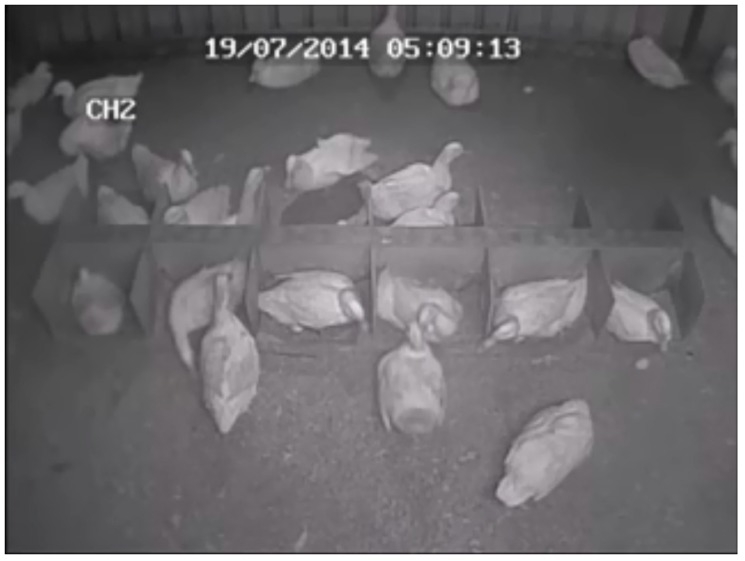
Still frame from video footage showing one group of 12 nest boxes used in the shed. A second group of the same layout and design is not pictured.

The study was conducted on a small commercial poultry farm in Western Australia. Fifty one female and nine male Pekin ducks from a breeding flock were housed overnight (7 p.m. to 7 a.m.) in one half of a fully enclosed shed (pen dimensions 5 m × 5 m, Figure 1), the other half being occupied by another breeding group. Once the shed door was opened each morning, the birds were free to move between the shed and an outdoor pasture-based range with an open water source. Birds were aged between 8 and 10 months at the start of the experiment and were not individually identified. They had been provided access to nest boxes from 18 weeks of age, to ensure familiarity with the boxes prior to the onset of laying. The nest-box ratio was approximately 1 box per 2 females. The boxes measured 0.4 m × 0.4 m and were open at the top and front (Figure 2). The nest boxes and shed floor were lined with sawdust (as is standard practice in many Australian duck farms), which the farmer topped up and cleaned each week. The ducks had ad-lib access to feed and clean drinking water at all times. The lighting schedule was maintained at 17 h light: 7 h darkness (9 p.m.to 4 a.m.), with artificial lighting used in the shed to extend the daylight period.

### 2.2. Video Recording and Quantification of Pre-Laying Behavior

A digital video recording system (Techview QV3034, Jaycar, Perth, Australia) was installed in the shed. Six cameras were attached to roof rafters in positions that ensured views of the entire shed floor would be recorded simultaneously. The ducks were recorded every day between 3 a.m. and 7 a.m. during the first 2 weeks of the month between May and August 2014. An ongoing technical issue during the recording weeks meant that the number of complete recordings of the entire 4 h period was limited to 6 in May, 5 in June, 8 in July and 5 in August, giving a total of 24 nights available for analysis. The decision to record between 3 a.m. and 7 a.m. was made based on a previous study of egg-laying in commercially kept Pekin ducks that found that the majority (65%) of eggs were laid in the hours after lights came on [16]. For the shed in this study, lights-on occurred at 4 a.m.; therefore, beginning recording at 3 a.m. allowed capture of the final hour of the dark period, and 3 h of light when peak egg-laying should theoretically occur, before the birds were released outside at 7 a.m.

For each of the 24 days, one incidence of floor-laying and one of nest-laying were identified. Floor-laying was defined as a bird depositing an egg directly onto the shed floor, not within a previously established nest that had been created in the floor substrate. This definition of floor-laying was used, as this is the working definition used by the Australian duck industry [18]. Eggs laid directly onto the floor also account for approximately 95% of eggs laid outside of nest boxes [19]; thus it was considered that analyzing the behavior of birds laying directly onto the shed floor would give a better representation of factors that might contribute to the incidence of the most common type of floor egg. Nest-laying was deemed to have occurred by identifying a nest box that was empty of eggs when a bird entered it, but with an egg present when the bird exited. Only the first egg laid in a next box was considered, as the presence of eggs in the nest could enhance nest box attractiveness [7] and thus be a confounder.

The duck’s behavior was analyzed for one hour prior to it selecting a site for oviposition. Video footage was reversed with video editing software (Avidemux video editor, version 2.5, https://avidemux.en.uptodown.com), to allow retrospective viewing of behavior. The period for behavioral analysis of nest-laying was taken from the time when the bird entered the nest box, to one hour beforehand. The period for behavioral analysis of floor-laying was taken from the time the bird was seen to lay an egg on the shed floor, to one hour beforehand. All behaviors observed in the one hour prior to oviposition site selection were continuously sampled and coded using behavioral analysis software (Interact, version 14.0, Mangold International, Arnstoff, Germany). Continuous sampling of all observed behaviors was achieved by simultaneously playing footage from the 6 cameras, so that the movements of each duck could be tracked between shed areas. The selection of each incidence of nest or floor-laying for each night was opportunistic, based on whether the duck could be reliably tracked on the video footage for the full hour before laying. In the event that the individual was lost in a camera view, or uncertainty arose as to whether the same individual was being observed, the entire observational period was discarded and another egg-laying event was selected for analysis. All behavioral observations and coding were performed by the same person.

Observed behaviors were considered as either state or events [20]. Behavioral states observed were walking, sitting, maintenance behaviors (drinking, eating, preening), or nest-building behavior (Table 1). Behavioral events were box entries, exits and investigations, occurrences of multiple birds in nest boxes, and social interactions (aggressive or non-aggressive; Table 1). Behavioral states were considered mutually exclusive; however it was possible for an event behavior to occur during a bout of state behavior (e.g., an aggressive interaction occurring while a bird was sitting). Additional behavioral variables (Table 1) were created post-hoc using the contingency analysis or co-occurrence functions of Interact.

Age was not considered to be a confounding factor across time, as all birds were well established layers at the beginning of the experimental period. The peak of floor-laying occurs in the early part of the laying cycle [1], with rates stabilizing at around 30 weeks of age [19], by which time birds are typically consistent in their nesting location preference [16]. As the flock age varied between 8 and 10 months at the start of the experiment, nesting behavior exhibited by individual birds was regarded as indicative for that bird.

### 2.3. Statistical Analysis

Data analysis was conducted using R statistical software [21]. All egg-laying events were assumed to be independent, although it is acknowledged that the lack of individual bird identification and unit replication are limiting factors. Exploratory analysis of the frequency, percentage of time, and duration was undertaken for each behavioral variable. Duration data used were the average bout lengths of behavior for each bird within the observational period. Data were visualized graphically to assist with the identification of trends in behavior between and within groups. This process was used to determine which behavioral variables were then subject to formal statistical testing. Consequently, maintenance and non-aggressive behavior was excluded from further analysis because of a low frequency of occurrence.

Chi-square tests were used to determine whether there were differences in the proportion of birds per group that engaged in walking, sitting, nesting-associated behaviors (nest-building behavior, nest box investigation, nest box entry), or aggressive encounters over the 1 h period.

The initial analysis of the frequency of nest box interactions identified that a sub-population of floor-laying birds did not enter or investigate nest boxes. This difference was included in further analysis by classifying the birds post-hoc into three groups instead of two: birds that laid on the floor but entered and investigated nest boxes before doing so (FL-In, n = 11); birds that laid on the floor and did not enter or investigate nest boxes (FL-Out, n = 13), and birds that laid in the nest boxes (NL, n = 24). Data sets could not be normalized by either log or square-root transformations, thus non-parametric analysis methods were used to determine if significant differences in the frequency, duration, or percent of time spent performing a behavior existed between groups. Further analyses used either Mann–Whitney U tests for comparing two groups (FL-In vs NL groups for behavior occurring in the nest boxes), or Kruskal-Wallis tests for three groups (FL-Out vs FL-In vs NL for behavior occurring out of the nest boxes). Post-hoc testing of significant Kruskal–Wallis tests used a pairwise Mann–Whitney U test with a Bonferroni correction. For all tests, the level of significance was *p* ≤ 0.05 and a trend was considered when 0.05 < *p* ≤ 0.1. Results presented for these analyses are median values with minimum-maximum ranges, unless otherwise stated.

A correlation of occurrences of multiple birds in the nest box versus the number of aggressive encounters had in the nest box was performed, to determine the strength of association between these variables.

## 3. Results

The locations of the 24 floor-laid eggs and the 24 nest-laid eggs for which behavior was analyzed are shown in Figure 1.

### 3.1. Nest Box Interactions

More nest-laying birds entered nest boxes in the hour prior to oviposition site selection than floor-laying birds (100% vs 46%, χ^2^ = 15.19, degrees of freedom (df) = 1, *p* < 0.001), with 13 of the 24 floor-laying birds never entering nest boxes (FL-Out). The frequency, percentage of time and duration of nest box entries did not differ between NL and FL-In groups (Table 2). Nest box investigations were performed by 100% of birds in the NL and FL-In groups (median number of investigations = 9 and 4, respectively, Table 2), but never by birds in the FL-Out group. The frequency, duration and percentage of time spent investigating nest boxes were not different between FL-In and NL (Table 2).

### 3.2. Nest-Building Behavior

Nest-building behavior was performed exclusively in nest boxes by NL. All of the FL-Out and one of the 11 FL-In ducks performed nest-building behavior on the floor. The remaining 10 FL-In performed nest-building behavior exclusively in the nest boxes. There were no significant differences in the frequency, percentage of time spent, or duration of nest-building behavior between the 3 groups (Table 2).

### 3.3. Behavior Outside of Nest Boxes

All birds engaged in bouts of walking. There were differences in the percentage of time spent walking between groups (H = 12.33, df = 2, *p* = 0.002); FL-Out spent less time walking than both FL-In (median value 2.8% vs. 21.1%, *p* = 0.001) and NL (median value 2.8% vs. 13.7%, *p* = 0.023). There was no difference in the frequency and duration of walking bouts between any of the 3 groups (Table 2). More FL-Out birds sat outside of the nest compared to FL-In (100% vs. 45%s, χ^2^ = 6.76, df = 1, *p* = 0.009), with a trend for more FL-Out birds sitting outside boxes compared to NL (100% vs. 71%, χ^2^ = 2.97, df = 1, *p* = 0.085) also present. There were differences in the number of sitting bouts between groups (H = 25.17, df = 2, *p* = <0.001), with FL-Out sitting more frequently than both FL-In and NL (median values 11 vs 0, p < 0.001 and 11 vs. 2.5, *p* < 0.001). The percentage of time spent sitting also differed between groups (H = 13.24, df = 2, p = 0.001), with FL-Out sitting more than both FL-In (median value 61.5% vs. 0.0%, *p* = 0.008) and NL (median value 61.5% vs. 19.5%, *p* = 0.019). There were also differences in the duration of sitting bouts (H = 7.75, df =2, *p* = 0.021), with FL-Out birds sitting longer per bout compared to FL-In (185.1 s vs. 0.0 s, *p* = 0.017).

### 3.4. Behavior Inside Nest Boxes

There was no difference in the percentage of time spent in nest boxes, or the duration of nest box entries between NL and FL-In birds (Table 2). There were no differences in the frequency, percentage of time, or duration of sitting bouts between NL and FL-In.

### 3.5. Aggressive Interactions

Every bird analyzed engaged in aggression in the hour prior to oviposition site selection. The total number of aggressive encounters differed between groups (H = 11.91, df = 2, *p* = 0.003), with FL-Out having fewer aggressive encounters than both FL-In (median value 6 vs. 35, *p* = 0.009) and NL (median value 6 vs. 30, *p* = 0.007; Table 2). Differences in the percentage of time spent in aggressive encounters were also identified (H = 10.12, df = 2, *p* = 0.006): FL-Out spent less time in aggressive encounters compared to FL-In (median value 0.61% vs. 5.6%, *p* = 0.02) and NL (median value 0.61% vs. 4.6%, *p* = 0.019; Table 2). The duration of aggressive bouts was not different between any of the 3 groups. The number of aggressive interactions initiated differed between groups (H = 12.26, df =2, *p* = 0.002), with FL-Out initiating fewer interactions compared with FL-In (median value 1 vs. 14, *p* = 0.007) and NL (median value 1 vs. 10, *p* = 0.02; Table 2). Differences in the number of aggressive interactions received were also found (H = 11.51, df = 2, *p* = 0.003), with FL-Out also receiving fewer aggressive interactions than FL-In (median value 5 vs. 21, *p* = 0.03) and NL (median value 5 vs. 24.5, *p* = 0.005; Table 2).

**Figure 3:**
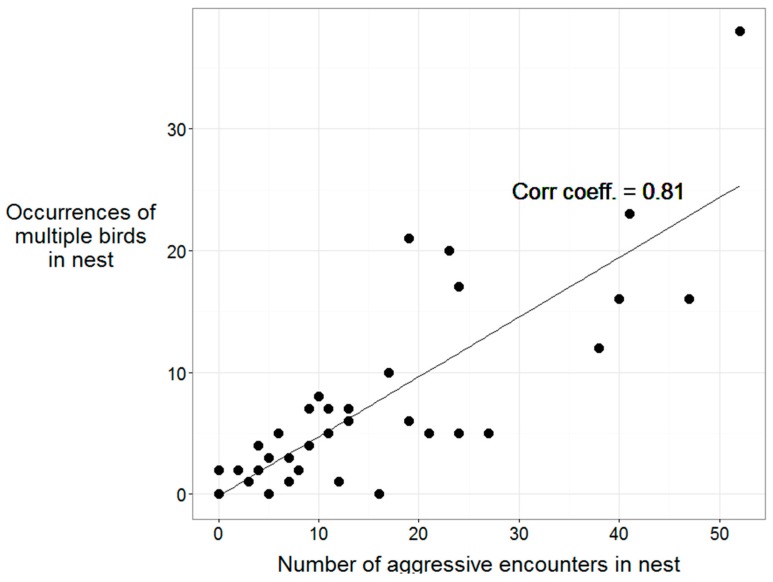
Correlation between the occurrences of multiple birds in the nest box and the number of aggressive encounters that occurred in nest boxes in all ducks that used nest boxes in the hour prior to oviposition site selection.

The percentage of aggressive encounters that occurred in the nest box were similar between FL-In and NL (Table 2). There were no differences between FL-In and NL in either the percent of total aggression that occurred in the nest boxes, or the percent of time spent in aggressive encounters when in the nest boxes (Table 2). Nest exits related to aggression accounted for 56.7% of all nest exits overall, with NL exiting the nest due to aggression 59.8% of the time, and FL-In exiting due to aggression 55.3% of the time. Exits from the nest boxes associated with either receiving or initiating aggression were not different between FL-In and NL groups (Table 2). There was a strong positive association between the occurrences of multiple birds being in the nest and the number of box exits due to aggression (R = 0.81, Figure 3).

## 4. Discussion

The aim of this study was to quantify the pre-laying behavior of nest-laying and floor-laying ducks in the hour prior to them selecting an oviposition site. It was hypothesized that differences would exist in the pre-laying behavior of floor-laying and nest-laying birds, and within the floor-laying population. The results indicate that nest box interactions, activity levels, and aggressive interactions were similar between some floor-laying birds and nest-laying birds. However, a sub-population of floor-laying birds that did not interact with nest boxes at all was identified (FL-Out). The FL-Out birds were less active and engaged in less aggression than the two groups of nest-using birds. The two sub-populations within the floor-laying ducks might have behaved differently due to differences in motivation due to effects of domestication, responses to competition for nest boxes, or nest design preferences. Consideration as to how these behavioral differences are linked to bird welfare needs to be given, as improved recognition of factors contributing to floor-laying in Pekin ducks provides industry with opportunities to develop practical management strategies that would result in both improved production and welfare outcomes.

An explanation for the differences in behavior seen between the two nest-using groups and FL-Out birds could be social hierarchy. Although it was not possible in this study to establish the flock hierarchy, because of the lack of individual identification, the impact of social hierarchy on floor-laying warrants mention. It could be argued that nest-layers are likely to be more dominant than floor-layers, as dominant birds often have priority access to important resources [22], such as a nest box. A clear distinction in dominance between nest-layers and FL-In birds is not supported by the results, as nest-layers and FL-In birds spent similar amounts of time in nest boxes and made a similar number of box entries. They also experienced similar levels of total aggression, nest-related aggression, and aggression-related nest exits, and did not initiate or receive aggression any more frequently. However, it may be possible that FL-Out birds are lower in the social order than both of these groups. Social hierarchy has previously been shown to alter the expression of pre-laying behavior in hens [23] with subject birds engaging in more avoidance behavior of dominant birds than subordinate birds when trying to access a nest site. Although floor-laying has not been associated with social rank in hens [22], further work with individually identified ducks is needed to examine the effect of social hierarchy on floor-laying in this species. However, any future demonstration of a relationship between floor-laying and social hierarchy in smaller groups of ducks (such as that seen here) may not easily be extrapolated to large-scale farms (often thousands of birds in a breeding shed). Stable hierarchies that are established through a series of dyadic encounters seem unlikely to occur in such large groups of ducks, as the relative costs of engaging with so many unfamiliar birds may be too great. This has been shown in chickens, where other social strategies that do not rely on individual recognition, such as status signaling are more likely to be used to avoid agonistic encounters in large groups [24]. How these alternative social strategies influence floor-laying in large groups has yet to be fully determined in any poultry species.

The lack of motivation to enter nest boxes in FL-Out could be due to the effects of domestication on energy expenditure [25]. It has been theorized that the expression of energy-demanding behaviors may have been coincidentally relaxed during domestication, as such behaviors are no longer as important for an animal’s survival [25]. Searching and competing for a nest site could be energy-expensive as the activities involved, such as locomotion (e.g., walking or running during aggressive chases) and defense of the nest box, require greater energy utilization [26]. The possibility that FL-Out birds are less motivated to seek out an appropriate nest site than the two nest-using groups is supported by the differences in the sitting and walking behavior. FL-Out birds spent more time sitting and less time walking than the FL-In and NL birds, suggesting that the latter two groups were more active in searching for a suitable oviposition site. The resource allocation theory suggests that animals undergoing intense selection for production traits will spend more time in energetically low-cost behaviors [25]; therefore the FL-Out birds may be electing to conserve energy by not engaging in the typical nest-seeking repertoire.

The finding that nest-building behavior differs only in location between the three groups of ducks indicates that, although FL-Out may not engage in the full range of pre-laying behaviors typically seen, they still exhibit some parts of the behavioral repertoire associated with nest site selection. It is possible that FL-Out birds do seek suitable nest sites within the shed environment, but that they do not consider the nest boxes attractive. Of the factors known to influence nest box use in chickens, the ones most likely to explain the disinterest shown for nest boxes by FL-Out are social factors and nest design. A lack of nest box experience is unlikely to explain floor-laying in this flock, as the ducks were well established layers that had at least three months’ experience with nest boxes before the start of the video recordings. In addition, the age of first nest box experience has been shown to have no significant effect on the proportion of floor-eggs in Pekin ducks [7].

A social factor that could contribute to FL-Out birds not using nest boxes is competition. The similar levels of engagement in aggression by FL-In and NL birds suggest that competition for the nest boxes exists in the pre-laying period. In contrast, FL-Out birds were engaged in fewer aggressive interactions, and spent less time involved in aggressive interactions than both other groups. A similar pattern has been found in chickens, where lower incidences of aggression occurred in hens that elected to lay away from the most preferred nesting site [27]. Perhaps not engaging with nest boxes is a coping strategy that FL-Out birds have adopted to avoid competition, thereby decreasing the amount of aggression (and by extension, stress) that they experience in their daily lives.

Competition for nest boxes also explains the ultimate egg-laying location of the FL-In birds. Aside from the similar levels of aggression between FL-In and NL birds, the strong correlation between occurrences of multiple birds in the box and the number of aggressive encounters in the box also indicates that competition for nest boxes exists. An impact of nest box competition on the occurrence of floor-laying in ducks has been described previously, with fewer floor-eggs occurring when the ratio of nest boxes to females was 1:1 compared with 1:4 [16]. Although the ratio of nest boxes to females in this study was high (1:2) by Australian industry standards, our findings indicate that competition for nests and subsequent floor-laying still exist at this allowance. These results lend further support to earlier studies that competition is a contributing factor to floor-laying in farmed Pekin ducks.

The occurrences of multiple birds in nest boxes raises the question of gregarious nesting behavior in Pekin ducks. Gregarious, or sociable, nesting in hens has been defined as a hen choosing to enter an occupied nest when an empty one is available [28,29]. Besides chickens, the behavior is presumed to exist in quail and turkeys, based on the presence of multiple eggs found in nest sites [30,31]. Gregarious nesting has also been observed in small groups of Pekin ducks [32]. The current study did not identify if a nest entry was gregarious (as per the above definition), but there is a stark contrast with the earlier findings in ducks: no aggression was seen when more than one duck occupied a nest box in the earlier study [32], whilst here the correlation between occurrences of multiple birds in the box and the number of aggressive encounters in the box is shown. This difference may be explained by the small groups used in the previous study (n = 8), as there was likely an established social hierarchy where all individuals were known to each other. As such, gregarious nesting may be better tolerated in that situation, compared with the larger flock size seen in this study, where perhaps less recognition of individuals results in aggression when one bird attempts entry of an occupied nest. It is as yet unclear what the origins of gregarious nesting in Pekin ducks are. Gregarious nesting in chickens has been proposed to occur as a result of social facilitation or local enhancement, particularly in inexperienced hens just coming into lay [29]. Whilst these phenomena may also be the case for Pekin ducks, a further explanation is that gregarious nesting may be derived from the behavior of brood parasitism seen in Mallard ducks nesting at high densities [33,34]. Although gregarious nesting is seen when the ratio of females to nest boxes is 1:1, the behavior occurs more often when the ratio is 1:4 [32]. This finding suggests that gregarious nest entries in Pekin ducks may, at least in part, have originated from brood parasitism strategies used by Mallards when competing for a preferred nest site. It is therefore possible that the aggression due to competition seen in this study has its roots in brood parasitism. Further work is required to better establish factors contributing to gregarious nesting in Pekin ducks, and how group size influences both its frequency and the type of social interactions that occur within nests.

Nest design, including structure and nesting substrate, could have contributed to the lack of interest in nest boxes shown by FL-Out. The boxes provided in the shed, although typical by Australian industry standards, are open at the top and front. The similar number of nest box entries, and the similar pattern of nest box investigations between FL-In and NL indicate that the level of enclosure provided by the boxes was considered the most adequate option available by these two groups. However, previous work has shown that Pekin ducks prefer a higher level of concealment than such open boxes provided [16]. It is thus possible that the FL-Out birds did not regard the level of concealment provided by the nest boxes as sufficient and found no additional benefit to using them.

Nesting substrate could be another factor of nest design affecting the motivation of FL-Out birds. In the present study, as in most commercial duck farms, sawdust was provided both in the nest boxes and on the shed floor. However it is possible either that sawdust is not the preferred nesting substrate for ducks or that the lack of contrast between the shed floor and the nest substrate might reduce the ducks’ inclination to use the next boxes. To our knowledge, the substrate preferences of Pekin ducks has not been investigated, but clear preferences have been demonstrated in hens and associated with the occurrence of floor-eggs [9,35,36].

It is also possible that the FL-Out birds in this study represent a persistent floor-laying sub-population, similar to that identified in previous work [16]. These authors found that approximately 5% of birds were persistent floor-layers. The design of the present study did not allow for a measurement of percentage of floor-laid eggs or percentage of floor-laying birds, and such data is not collected by the producer. Nevertheless, this study demonstrates that two sub-populations exist within the floor-laying group: one engages with nest boxes, while the other does not. It would be useful to determine if the birds showing no interest in nest boxes are persistent floor-layers.

The results of this study give cause to consider what the welfare implications of floor-laying in Pekin ducks may be. It is reasonable to suggest that nest site competition and the related aggression that exist in FL-In and NL birds is a negative experience, as aggression results in fear and distress [3]. The increased walking seen in these two groups may also be a sign of frustration, as they actively seek and compete for nest sites. Further work is required to quantify any stress response and establish the cost to those birds. Whether FL-Out birds experience stress or negative emotional states such as frustration also needs to be established. FL-Out birds exhibited increased sitting behavior in the hour prior to egg-laying. In chickens, sitting behavior is typically considered an indicator of settled nesting behavior [37], whilst increased locomotion is thought to be indicative of unsettled nesting behavior, which may in turn lead to frustration [38]. However, increased frequency of sitting behavior with reduced total sitting time has previously been correlated with elevated plasma corticosterone, suggesting unsettled nesting behavior with an associated stress response [39]. The sitting behavior exhibited by FL-Out birds in this study may represent a pattern of settled behavior in birds with low motivation to seek out an enclosed nest. Alternatively, the increased sitting may be the external manifestation of these birds’ coping or avoidance behavior. Therefore, more study is needed to determine the significance of increased sitting in behavior in ducks that do not use nest boxes.

The distribution of FL-Out oviposition sites (Figure 1) indicate that they do not seek out other enclosed areas in the shed, away from the contested nest boxes (e.g., in corners or against walls). Rather, the frequent occurrence of floor-eggs near the drinker indicates that this is the most likely area for floor-laying birds to lay an egg. This theory is supported by the producer’s own observations of where floor-eggs are typically found each day [40]. One possible explanation is that floor-laying birds are opting to engage in drinking behavior as a displacement activity at or near the time of oviposition. If a behavior that an animal is highly motivated to perform is thwarted, displacement behaviors, such as drinking [41] or preening [42] may occur due to frustration. Although not quantified in the current study, it would be useful to further investigate the association between floor-laying and displacement activities such as drinking or preening, to better establish the welfare implications of floor-laying in ducks.

A significant limitation of this study was the inability to identify individual birds for the duration of the filming period. Although all observations were considered independent of one another, it is possible that the same bird may have been analyzed more than once, potentially resulting in misrepresentation of behaviors within and between groups. However, the probability of the same bird being observed on any two nights is relatively low, at approximately 4 in 10,000. Given that this study was based within a commercial operation, it was not feasible to retain individual identifiers that were camera-visible for the entire filming period. The limitations of the data analysis are recognized, but these initial results do provide evidence of differences in Pekin duck pre-laying behavior that can be used to inform future larger scale studies.

A further limitation of this study was to use only two categories for location, either in or out of the nest boxes. Most birds were observed to move through a very restricted area of the shed and spent the majority of time near one set of nest boxes or one particular area of the shed floor. However, it would have been informative to be able to quantify the time spent at different distances from the nest to better understand the level of interest shown for nest boxes by individual birds, and the relationship between nest proximity and levels of aggression experienced.

## 5. Conclusions

This study identified that some floor-laying Pekin ducks never engage with nest boxes in the hour prior to selecting a laying site, whilst some floor-layers use nest boxes similarly to nest-laying birds. Competition for nest boxes, as indicated by aggressive interactions, appears to be an important contributing factor. However, other factors that might affect birds’ willingness to compete for a nest box, such as nest attractiveness, should be considered. The results of the present study indicate that an industry focus on practical strategies to reduce competition could help mitigate floor-laying. This in turn would assist in improving the production efficiency of these systems, and decreasing any negative welfare outcomes that might exist.

## Figures and Tables

**Table 1:** Ethogram used for statistical analysis. Primary observations are those behaviors that were directly observed from the video footage of the hour prior to oviposition site selection. Secondary variables were created using either the contingency analysis or co-occurrence function of Interact software.

Behavior	Description
**Primary Observations**	
*States:*	
Walking	Duck is outside of the nest box, moving around the shed floor.
Sitting	Duck is sitting down quietly with wings tucked in, either awake or asleep. Occurs either inside or outside of the nest box.
Maintenance behavior	Duck is engaged in either feeding, drinking or preening. Each of these is further defined as: duck is standing at the feeder actively engaged in bouts of food consumption; duck is standing at the drinker, actively engaged in bouts of drinking; duck is engaged in self-directed grooming/cleaning of body.
Nest-building behavior	Duck is using body and feet to create a hollow in the substrate and/or manipulating substrate with beak in the nest hollow. Occurs either outside nest boxes on the floor, or inside a nest box.
*Events:*	
Nest box entry	Duck places full body inside a nest box.
Nest box exit	Duck places full body outside a nest box.
Nest box investigation	Duck stands outside of nest box with neck extended and head inside box, may or may not engage with box substrate.
Multiple birds in nest box	Subject bird plus 1 or more birds with full bodies in the nest box concurrently.
Aggressive behavior	Subject duck is engaged with other bird/s, overt aggression (e.g., grabbing neck skin, feather pecking, chasing) is seen. Subject either receives or initiates aggression.
Non-aggressive behavior	Subject duck is engaged with other bird/s (e.g., sitting in physical contact with another bird, permitting or performing investigative behavior from/towards another bird as passing by), but no overt aggression is seen. Subject either receives or initiates non-aggressive behavior.
**Secondary Variables**	
Time in nest boxes	Duration of nest box visits, calculated between occurrences of nest box entries and exits.
Aggression in nest box	Event where subject duck was concurrently in the nest box and involved in aggressive encounter. Subject either receives or initiates aggression.
Nest box exit due to aggression	Event where a duck involved in an aggressive encounter co-occurred with that duck exiting the nest box. Subject may either be initiating or receiving aggression.

**Table 2:** Frequency, percentage of time spent and duration (s) of behaviors performed by laying Pekin ducks in the hour prior to oviposition site selection. FL-Out = ducks that floor-laid and did not enter nest boxes; FL-In = ducks that floor-laid but did enter nest boxes; NL = ducks that laid in nest boxes. Values are median values, with the minimum and maximum range in brackets. Values with different superscripts across rows are significantly different (*p* ≤ 0.05). Values with * across rows show a trend towards significance (0.1 ≤ *p* > 0.05).

Behavior	FL-Out (n = 13)		FL-In (n = 11)		NL (n = 24)	
	Median	(Min–Max)	Median	(Min–Max)	Median	(Min–Max)
**Nest box entries**						
Frequency	0	(0–0)	10	(2–15)	7	(1–26)
Percent time	-	-	46.45	(29.5–78.2)	47.29	(5.19–86.9)
Duration	-	-	142.1	(96.5–311.1)	149.6	(374.0–1535.0)
**Nest box investigations**						
Frequency	0	(0–0)	4	(0–28)	9	(0–30)
Percent time	-	-	0.3	(0.0–3.4)	0.9	(0.1–7.5)
Durations (s)	-	-	2.0	(0.0–7.2)	3.0	(1.4–9.0)
**Nest-building**						
Frequency	8	(1–24)	7	(3–12)	6	(0–23)
Percent time	10.8	(1.4–13.0)	6.5	(1.6–9.2)	3.9	(0.0–14.1)
Duration (s)	31.9	(15.5–89.4)	31.2	(19.5–49.0)	22.3	(0.0–63.8)
**Walking**						
Frequency	25	(2–91)	26	(10–57)	19	(3–103)
Percent time	2.8 ^ab^	(0.4–13.4)	21.1 ^a^	(8.5–47.0)	13.7 ^b^	(1.1–33.5)
Duration (s)	7.9	(6.8–9.3)	5.9	(3.8–11.0)	6.8	(3.6–10.0)
**Sitting outside box**						
Frequency	11 ^a^	(7–28)	0 ^b^	(0–4)	2.5 ^b^	(0–14)
Percent time	61.5 ^a^	(22.4–86.0)	0.0 ^b^	(0.0–38.7)	19.5 ^b^	(0.0–71.0)
Durations (s)	185.1 ^a^	(57.1–328.6)	0.0 ^b^	(0.0–200.1)	80.4 ^ab^	(0.0–391.6)
**Sitting inside box**						
Frequency	-	-	7	(0–19)	7	(0–23)
Percent time	-	-	34.9 *	(9.8–66.3)	50.6 *	(0.0–86.8)
Duration (s)	-	-	81.3	(51.9–198.7)	115.0	(77.4–390.1)
**Aggressive interactions**						
Frequency	6 ^ab^	(2–44)	35 ^a^	(12–87)	30 ^b^	(2–112)
Percent time	0.61 ^ab^	(0.0–4.5)	5.6 ^a^	(2.2–11.2)	4.6 ^b^	(0.6–14.1)
Duration (s)	2.8	(0.0–6.2)	14 ^a^	(2–43)	10 ^b^	(0–52)
Number initiated	1 ^ab^	(0–27)	14 ^a^	(2–43)	10 ^b^	(0–52)
Number received	5 ^ab^	(2–33)	21 ^a^	(10–44)	24.5 ^b^	(2–85)
% Total aggressive encounters occurring in nest box	-	-	44.4	(25.0–93.6)	33.9	(0.0–83.3)
% Total aggression occurring in nest box	-	-	5.6	(2.1–9.7)	7.6	(1.0–15.1)
% Time in aggression when in nest box	-	-	52.5	(28.9–94.1)	53.1	(16.0–89.0)
Aggression associated nest exits						
Total	-	-	7	(1–10)	4.5	(0–27)
Aggression initiated	-	-	2.5	(1–4)	2	(1–5)
Aggression received	-	-	5	(2–9)	5	(1–22)
